# SOSHI-seq: a high-throughput screening assay to test the functionality of putative response elements for nuclear hormone receptors

**DOI:** 10.1038/s41598-025-29970-8

**Published:** 2025-12-06

**Authors:** Shijia Wu, Romain Guyot, Yanis Zekri, Wenzheng Jiang, Frédéric Flamant

**Affiliations:** 1 Ecole Normale Supérieure de Lyon, INRAE, CNRS, Institut de Génomique Fonctionnelle de Lyon, 69364 Lyon Cedex07, France; 2 Present Address: Divisions of Oncogenomics, The Netherlands Cancer Institute (NKI), Plesmanlaan, 121 1066CX Amsterdam, Netherlands; 3 Shanghai Key Laboratory of Regulatory Biology, School of Life Sciences, East China Normal University, Shanghai, China

**Keywords:** Deep sequencing, Nuclear receptors, Thyroid hormone, Gene regulation, Genomic analysis, High-throughput screening

## Abstract

We present a cheap, rapid, and versatile assay called SOSHI-seq (Screening of Self-transcribed Hormone Inducible response elements coupled to sequencing). The protocol is an adaptation of the STARR-seq method, which allows testing the capacity of thousands of synthetic DNA sequences to act as hormone-response elements. Using the nuclear receptors of thyroid hormone as an example, we show that SOSHI-seq is a suitable complement to ChIP-Seq analysis to identify at genome-wide scale the functional response elements occupied by nuclear receptors in chromatin.

**Supplementary Information:**

The online version contains supplementary material available at 10.1038/s41598-025-29970-8.

## Introduction

Nuclear receptors (NRs) form a large class of transcription factors that can bind directly to DNA and regulate the transcription of genes upon ligand binding^1^. The 48 human nuclear receptors are present in many cell types and play important roles in development, homeostasis, and metabolism. They are also involved in many pathological^2^ and evolutionary processes^3^. Therefore, the identification of the genes which transcription is directly controlled by the NRs, or NR target genes, is an important task, which is pursued for many cell types in various animal species. These target genes are usually defined as genes which expression is quickly down- or up-regulated after ligand addition, and which regulatory sequences are occupied by the associated NR.

The DNA consensus sequences for the binding of NR are known, and the vertebrate genomes contain many of them (> 50 000 for a given NR in a vertebrate genome). However, their occurrence near ligand-responsive genes is not sufficient to infer the actual binding of the NR because chromatin accessibility is regulated in a cell-type-specific manner. Thus, chromatin occupancy by NR is a key parameter, which must be defined experimentally in each cell type. The definition of this so-called cistrome is currently achieved by ChIP-seq analysis. However, experience proves that combining transcriptome and cistrome datasets to define NR target genes is difficult. It is generally observed that the number of NR binding sites in chromatin exceeds by far the number of genes whose transcription level is sensitive to the NR ligand. This implies that many NR chromatin binding sites do not mediate a ligand-dependent transcriptional response, which can receive at least two types of explanation. First ChIP-seq might capture NR/DNA interactions which are too labile for efficient cofactors recruitment. Second, many of the occupied genomic sites do not contain known consensus sequences. This can be explained by the presence of intermediate DNA-binding proteins, which tether NR to DNA, a configuration that does not necessarily promote a ligand dependent regulation of transcription. Overall, it seems that the binding of NR to chromatin is necessary but not sufficient to mediate a ligand-dependent transactivation. Therefore, a better definition of NR target genes requires defining whether the chromatin binding sites are functional and act as genuine ligand response elements.

We propose here a simple adaptation of the STARR-seq method, which was previously developed to identify transcription enhancer at genome wide scale^4,5^. STARR-seq is usually a laborious and difficult procedure used to screen DNA libraries for enhancer activity^6^. In this simplified version, called SOSHI-seq (Screening Of Self-transcribed Hormone Inducible response elements coupled to sequencing) a pool of long oligonucleotides carrying putative DNA response elements is produced by chemical synthesis, cloned in a STARR-seq vector and transfected in cells, together with vectors for NR expression. Because the putative response elements are inserted in a transcribed portion of the STARR-seq vector, RNA analysis enables to precisely evaluate the ligand response of each cloned elements. We present proof of principle experiments performed for the thyroid hormone receptors TRα1 and TRβ1, which demonstrates that this SOSHI-seq protocol is a low-cost, versatile and rapid assay suitable for high throughput screening of putative response elements.

## Results

### Experimental setting

**Fig. 1:**
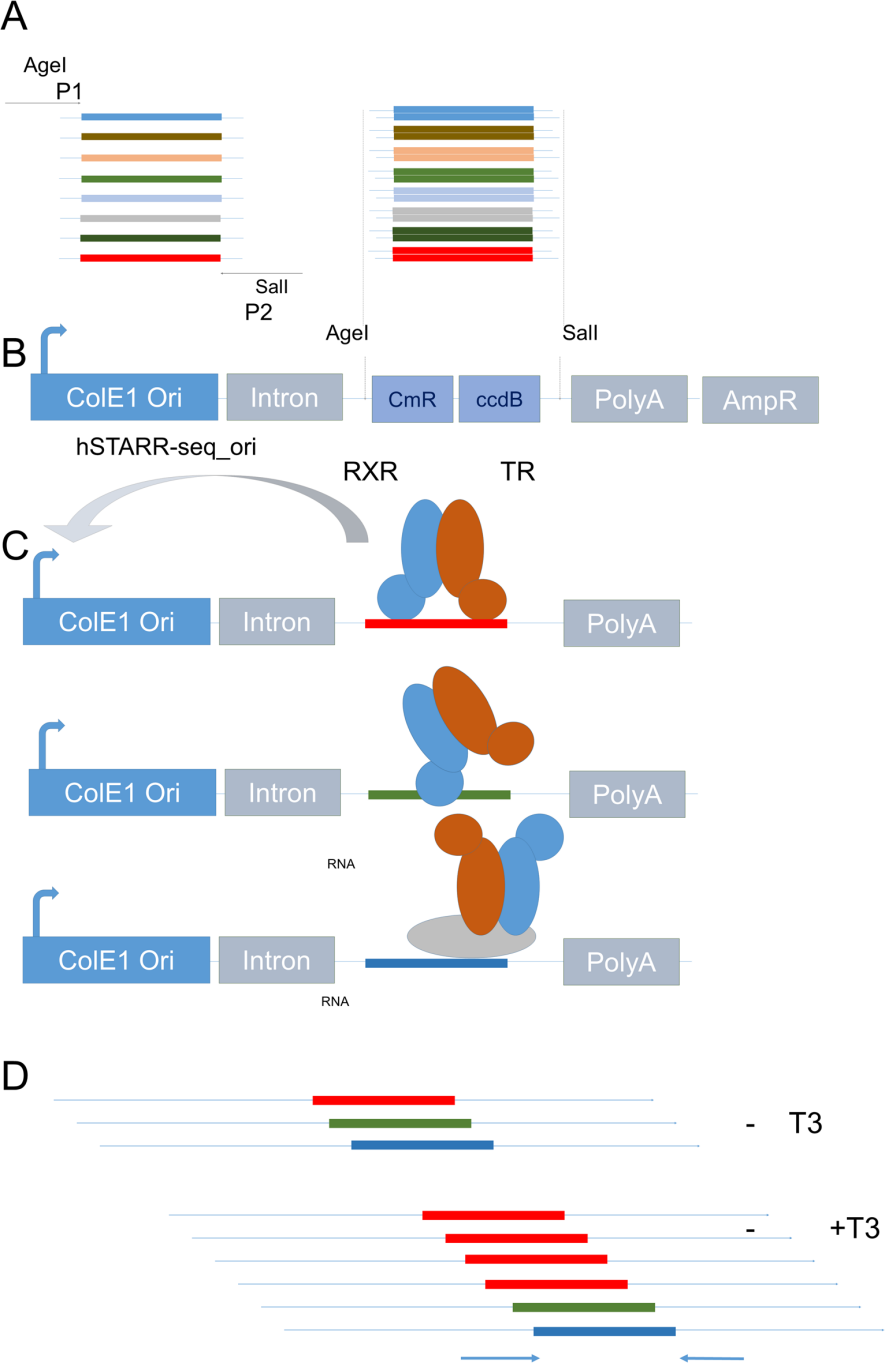
General SOSHI-seq procedure to screen DNA fragments with putative hormone responsive elements, applied to TRs. (**A)** Synthesis of a pool of 1000 oligonucleotides with putative binding sites for TRα1 or TRβ1 (TR). The nucleotides located around the peak summit are synthesized as an oligopool, with adapters on each side. The oligopool is converted to double-stranded DNA and amplified by low-cycle PCR (P1 and P2 primers) (**B**) The amplicon is digested with the AgeI and SalI restriction enzymes, purified by gel electrophoresis and cloned in the transcribed portion of the pSTARR-seq_ori vector. (**C**) Library DNA is cotransfected in human HEK293 cells together with pSG5-RXRα + pSG5-TRα1 or pSG5-RXRα + pSG5-TRβ1. Three examples of interaction of TR/RXR heterodimer and DNA, as it can be captured by ChIP-seq analysis: in the first example, the red sequence corresponds to a high affinity binding , which enable the T3 mediated transactivation of the pSTARR-seq_ori minimal promoter. In the second case, the interaction with green DNA sequence is weak, and not sufficient for transactivation. In the third case, TR/RXR is tethered to the blue DNA fragment by protein/protein interactions and cannot transactivate. (**D**) Cells are cultured with or without T3 (10^−8^ M) before RNA extraction. The cloned inserts are amplified from cDNA with specific primers before deep sequencing. Read counts are computed and used for statistical analysis. In the shown example, the sequence colored in red is the only one to mediate a T3 dependent transactivation and is overrepresented in the RNA of T3 treated cells.

Thyroid hormone (3,3′,5-triiodo-L-thyronine, or T3) acts by binding to two highly similar NR, TRα1 and TRβ1. The two TRs form heterodimers with a RXR receptor. TR/RXR heterodimers preferentially bind a consensus DNA motif with two half-sites separated by a 4 nucleotides spacer (consensus DR4: 5′AGGTCANNNNAGGTCA3′)^7^. Figure 1 outlines the main step of SOSHI-Seq that we used to test the capacity of genomic sequences to mediate T3/TRα1 and T3/TRβ1 response. We started by selecting 1000 TRα1 binding sites (TRBS) in the mouse genome gathering a subset of the TRα1 cistrome previously defined in striatum neurons^8^. Fragments were randomly picked in these datasets (GEO Accession: GSE210975 and GSE143933) to include TRBS located in different regions (upstream or downsteam of transcription start site, 5′ untranslated regions, introns, unannotated) and with different properties (with or without DR4, proximal to T3 responsive genes or not). The sequences were synthetized as a pool (Fig. 1a) and cloned in the pSTARRseq-ori vector^9^ (Fig. 1b). The whole library, called library 1, was introduced in competent *E. coli*, and DNA was directly extracted from the bacterial colonies. We then co-transfected the library DNA with expression vectors (400 ng pSG5-RXRα + 100 ng pGL2DR4luc + 1500 ng of library DNA /well + 400 ng pSG5-TRα1 or pSG5-TRβ1) to ensure the presence of either TRα1/RXRα or TRβ1/RXRα heterodimers in the transfected HEK293 cells, which barely contain nuclear receptors (Fig. 1c). Transfected cells were treated or not with T3 (10^−8^ M; 24 h) before RNA extraction. The pGL2DR4luc construct was introduced to perform a control of luciferase activity in a fraction of the transfected cells (Suppl. Fig. S1b). Because the putative T3 response elements are introduced in the transcribed portion of the plasmid vector, we expected that the frequency of the active sequence in the RNA extracted from the transfected cells would be selectively increased by the T3 treatment (Fig. 1d).

### Identifying fragments driving T3 response

**Fig. 2:**
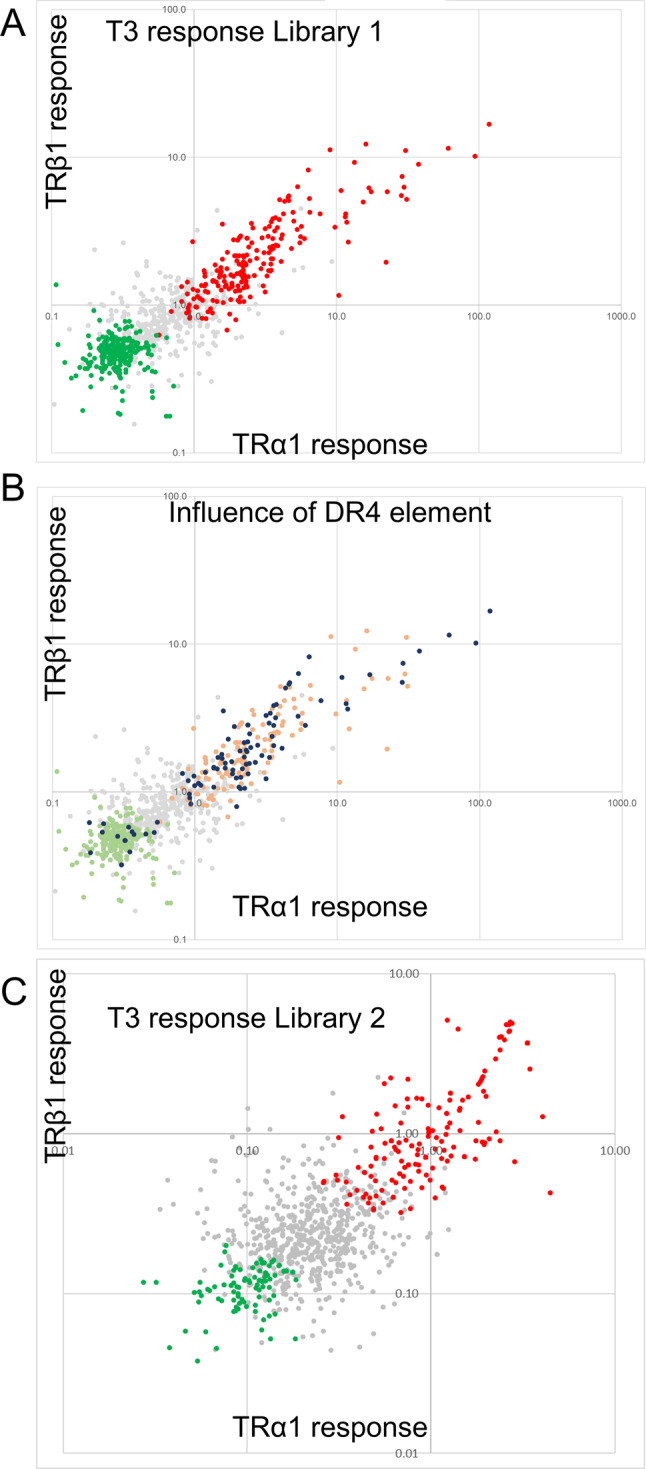
T3 mediated response by genomic fragments according to SOSHI-seq. (**A**) Fold-changes measures for TRα1/RXRα heterodimers (x-axis) or TRβ1/RXRα heterodimers for Library 1. Each dot corresponds to a distinct DNA fragment cloned from the oligopool. Differential statistical analysis (Deseq2) identifies DNA fragments which promote T3 mediated transactivation (red) or transrepression (green). (**B**) Same data. The active fragments (green or orange) which contain a DR4 like element (p-value < 10^–6^ according to FIMO analysis) are colored in dark blue. DR4 are enriched in the set of fragment promoting T3 transactivation (2.1 enrichment; χ^2^(5, N = 835) = 51.0; p < 10^–5^) (**C**) Same analysis as A for library 2.

Deep sequencing was used to sequence the fraction of RNA derived the library inserts. Raw sequences (> 5.10^6^ reads/sample) were aligned on the mouse genome to determine the number of reads corresponding to the selected genomic fragment (Suppl. Table S1). If one considers only the 800 genomic fragments for which at least 10 reads were found in each sequencing file, the fold-changes (with T3 vs. without T3) of the fragment frequency (reads per millions; rpm) widely varied, ranging from 0.1 to 55. For each fragment, the correlation between the fold-changes induced by either TRα1/RXRα or TRβ1/RXRα was high (R^2^ = 0.74). This result was also highly reproducible as we observed a high correlation (R^2^ = 0.85) between the fold-changes calculated in this experiment and the ones obtained in another experiment performed with a new batch of cell, new plasmid preparations, and a different sequencing run (Suppl. Table S1). This repetition and the assumption that TRα1 or TRβ1 has similar transactivation capacity for most DNA fragments, allowed to perform a statistical differential analysis (DESeq2, 2 factors, taking into account a possible batch effect) to highlight 244 DNA fragments which mediate either positive or negative T3 response (Adjusted p-value < 0.05. Fold-change > 1 or < − 1) (Fig. 2a).

Because SOSHI-seq only provides relative values, the observed fold changes do not immediately translate into transactivation or transrepression capacity. For example, a library containing only fragments which would be equally efficient at transactivation would only lead to fold changes close to 1. Therefore, the analysis only ranks the DNA fragments by their capacity to regulate transcription. We performed a quantitative PCR to evaluate the global change in library RNA induced by T3, which indicates that T3 only has a modest influence on the overall cellular content of library derived RNA (Suppl. Fig.S1b).

To confirm in an independent manner that the observed effects of T3 result from the regulation of transcription, we cloned 11 of the fragments present in the oligopool in the upstream regulatory sequence of the pGL2-derived luciferase reporter construct. The sequences were chosen to represent 3 typical situations: high positive response to T3, preferential activation by TRβ1, and downregulation by T3. We analyzed the T3 response of the luciferase activity of these constructs, and compared the response obtained using pGL2-DR4luc as a positive control. The results provide an independent confirmation that some the tested fragments mediate a very robust T3 response, and that liganded TR can mediate a negative transcriptional response (Table 1). We also verified that some fragment display a preferential response to TRβ1.

**Table 1:** Correlation between SOSHI-seq and luciferase based assay.

Fragt number	Distance TSS (kb)*	Fold-change TRα1 (luc assay)	Fold- change TRβ1 (luc assay)	Fold-change TRα1 (SOSHI-seq)	Fold-change TRβ1 (SOSHI-seq)
114	+ 101.7	126.8	104.7	34.4	45.8
142	+ 22.5	0.3		0.3	
194	+ 0.2	2.5	0.97	1.5	1.0
199	− 45.3	109.0		28.5	
318	− 12.8	0.2		0.1	
402	− 4.5	8.9		9.8	
429	0.4	86.9	82.0	45.3	55.3
484	− 5.2	20.5		1.5	
707	− 99.6	480.1		1.3	
832	+ 2.7	3.4		14.7	
955	− 17.8	0.2		1.8	
5xDR4		507.2		227.3	

*Transcription start site. Positive values means downstream to TSS.

We then mapped the active response elements by introducing point mutations. This firmly established that the positive response to T3 is mediated by the identified DR4 element in the Lib1-429 DNA fragment (Suppl. Fig. S2a). Interestingly the negative response element present in the *Thra* gene mapped to a splice donor site of the *Thra* gene (Suppl. fig. S2B). The preferential response TRα1 or TRβ1 of a DNA does not seem to rely on DR4 sequence itself as it is only observed when flanking sequences are present (Suppl. fig. S2C).

We addressed the influence of different parameters on T3 response, searching for criteria that would be predictive of transcriptional activity. As expected, the presence of the DR4 like consensus sequence proved to be correlated with the capacity to drive T3 activation (Fig. 2B). Although this was less visible, we also found that the largest peaks, as defined by previous ChIP-seq analysis in striatum were more likely to contain active fragments. This conclusion was reached by addressing the distribution of fragment which have the highest fold-change over local background (> 70 according to the MACS2 callpeak software for the GSE143933 dataset) among the 3 categories of fragment (up-regulated, down-regulated or not regulated. χ^2^(5, N = 835) = 19.5 p < 0.01). There are however active fragment in peaks with much lower signal to noise ratio, and this parameter can hardly be used to predict the capacity of genomic fragments to transactivate. Although many of the fragment mediating T3 activation originated from introns, the position of the fragment with respect to gene components, or their distance to the transcription start site did not correlate with their activity.

### Screening for active DR4 in ChIP-Seq datasets

The previous experiment indicates that TRBS, as defined by ChIP-seq, are more likely to provide a positive response in the SOSHI-seq assay if they contain a DR4 element. We also failed to identify another consensus DNA sequence predictive of activity. We thus constructed a novel library from another oligopool designed to address the activity of all the known TRBS with a DR4 element, as compiled in a recent review^10^, which were identified using the FIMO algorithm^11^. We first listed the 747 DR4 elements located within 30 kb of the transcription start site of a gene, for which ChIP-Seq analysis demonstrates the occupancy by TRα1 or TRβ1 in at least two of the following mouse cell types: cardiomyocytes, brown adipocytes, hepatocytes and GABAergic neurons of the striatum. We added to these 747 DR4 elements, 246 additional TRBS with a DR4 element for which a T3 response coincides with TR occupancy in at least one mouse tissue. This new oligopool was used to construct library 2. As the previous experiments showed that some large oligonucleotides may be difficult to amplify and clone, we restricted the size of the synthetized oligonucleotides to encompass only 108 nt of genomic sequence. Sequencing depth was sufficient to evaluate the activity of 936 fragments (minimum number of reads > 10). The experiment was also duplicated with TRβ1 to allow for differential expression analysis, which identified a supplementary set of 221 genomic DNA fragments able to mediate a T3 response in the SOSHI-seq assay (Suppl. Table S2 and Fig. 2C).

## Discussion

The SOSHI-seq protocol that we designed represents a novel and versatile tool to explore the functions of nuclear receptors in vitro and to identify functional hormone-responsive elements. Its aim is thus different from the one of STARR-seq, which is suited to identify constitutive transcription enhancers. Focusing of hormone-induced fold changes greatly simplifies the protocol. First, the use of an oligopool appears to be a cheap and convenient alternative to the tedious preparation of libraries, previously based on either direct cloning of genomic DNA or large-scale preparation of individual PCR fragments. Overall, the SOSHI-seq could be easily scaled up, as oligopools can be synthesized with up to 10,000 fragments of 300 nucleotides if necessary, covering close to 3 Mb.

The other simplification offered by SOSHI-seq is that it considers only ligand-induced fold changes. This overcomes a number of limitations and biases inherent to the STARR-seq principle. In particular, stabilization of mRNA by the inserted sequence^12^ has the same effect on mRNA abundance as a transcriptional enhancement in STARR-seq experiments. The introduction of splicing signals might also lead to the elimination of part of the transcript sequence. There is thus no need to use sophisticated methods to correct the biases caused by the uneven cloning efficiency of the DNA fragments^13,14^.

We found that around 30% of the TRBS selected from the ChIP-seq data were active in SOSHI-seq, outlying the necessity to complement chromatin analysis by a functional screening. This observation can receive several explanations. The first one is a technical limitation of ChIP-seq, which has limited spatial resolution, notably for peaks of small size. While defining the genomic window that is closest to the peak summit, we may have missed some active DNA motifs. This limitation might be more apparent for library 2, which contains smaller DNA inserts. Although very short fragment are active in this assay^7^, it might be advantageous to increase the fragment size in future experiments, which generally display stronger enhancer activities^6^. A more likely alternative explanation for the absence of T3 response from many TRBS would be that TR recruitment to chromatin is not always triggering a transcriptional activation. In the present situation, we tested many TRBS which do not possess a consensus motif known to enable the DNA-binding of TR/RXR heterodimers, and these fragments were often inactive. It is however more likely that TRBS identified by ChIP-seq often reflect TR tethering, i.e. the recruitment of TR to chromatin through protein–protein interaction. Previous work notably identified CTCF as a DNA binding protein able to interact with TR and tethers it to sequences which are not mediating T3 response^15^. Mining available datasets we found for example that almost 10% of the TRBS in mouse liver overlap with CTCF binding sites, which is consistent with the tethering hypothesis. We also often observe in ChIP-seq experiments the presence of several TRBS around T3 responsive genes, among which only one possesses the consensus DR4 element. We thus believe that TR participate to the formation of multiprotein complexes which contact chromatin at several neighboring sites, all captured by ChIP-seq analysis, among which only one contains a sequence active in SOSHI-seq.

The negative regulation of transcription by liganded TR that we observed is surprising, and further investigation is needed to address its possible relevance to the in vivo situation. While TRBS are enriched near T3-activated genes, they are not often located near T3-repressed genes. We previously interpreted this as an indication that negative regulation is not directly mediated by TR, but secondary to activation of transcription repressors^8,15^. However, different TRBS recruit transcription activators and repressors differently, leaving open the possibility that binding of TR/RXR heterodimers to DR4 sometimes leads to transcriptional repression^16^. Here, most of the fragments (88% in library 1) that showed a negative T3 response did not have a DR4 consensus, which suggests that the negative regulation depends on a distinct mode of binding of TR to DNA. However, the search for consensus motives failed to identify a sequence enriched in the fragments mediating transrepression. Furthermore, none of the genomic fragments capable of mediating transrepression is proximal (< 30 kb) to a transcription start site of a gene that is down-regulated by T3 in at least one cell type. Interestingly, there is one exception, which is a negative regulatory element originating from the *Thra* gene itself, which encodes TRα1. The negative response element was precisely mapped at an alternate splicing site, which is known to produce the non-receptor isoform TRα2^17^. When present in large excess TRα2 has the capacity to antagonize the transactivation exerted by the liganded TRα1^18^. The overlap between a negative response element to T3 and the alternate splicing site adds a possible level of regulation of the cellular response to T3, which remains to be investigated.

In conclusions, SOSHI-seq appears as a simple and versatile tool to study NR function. It proves to be a suitable complement to ChIP-seq analysis to identify the genes which transcription is regulated by liganded NRs. It is also a versatile assay which could be used to address other questions, like the possible competition between nuclear receptors, the selective regulation exerted by synthetic compounds, etc.

### SOSHI-seq limitations

In our experiments, we did not identify the causes for the absence of a small fraction of the synthesized sequences in the final datasets. This absence might reflect possible biases at each step of the protocol: oligosynthesis, PCR amplification, cloning, transcription, and reverse transcription. Coverage would probably be improved by increasing the number of bacteria colonies used for DNA preparation.

One limitation of the current protocol is that it does not provide absolute quantification, as we cannot perform a precise correction from variations in the total amount of library-derived RNA, which increases after T3 induction. Introducing several internal controls into the SOSHI-seq libraries would allow for a more precise calibration, notably when a large fraction of the library fragments is active. These could include fragments without binding site as negative controls, and several reference fragments from well characterized responsive fragments as positive controls. Additional spike-in controls could also be considered to facilitate comparisons across libraries and repetitions. Furthermore, the SOSHI-seq remains an in vitro assay in which NRs exert a regulation on transfected DNA, which represents a poorly chromatinized substrate, and thus might not faithfully model the diversity of in vivo regulations by NRs.

## Material and methods

All methods were carried out in accordance with relevant guidelines and regulations.

### Libraries construction

Customized pools of 1000 200-mers (library 1) or 150-mers (library 2) were produced by chemical synthesis (Twist Oligo Pools; Twist biosciences, South San Francisco) which associate genomic sequences and common flanking sequences. The central nucleotide sequence (158 or 108 nucleotides) was copied from mouse genomic sequences (Suppl. Table S1 and Suppl. Table S2. N.B: 8 fragments with sequences errors have been deleted from each table and omitted from later analysis). The remaining flanking sequences (5′ ATACTAGTCGCACTACGATC…. ACACCTAATGCAGAGCTAGCCA3′) were introduced to allow for global PCR amplification. 20 ng of the oligopool were amplified (forward primer: 5′GGCTAACCGGTGCTAGCATACTAGTCGCACTACGATC3′; reverse primer 5′TGAAAGTCGACGCTAGCT CTGCATTAGGTGT3′ 12 PCR amplification cycles were performed as follow: 95 °C for 30 s, 58 °C for 30 s and 72 °C for 30 s (GoTaq® DNA Polymerase). PCR products were purified on column (NucleoSpin® gel and PCR Clean-up; Macherey Nagel) and recovered in Smart Buffer (New England Biolabs) for restriction digestion (1 h, 37 °C, SalI-HF + AgeI-HF; New England Biolabs; restriction sites underlined in the PCR primers). The resulting PCR products, containing a mixture of DNA fragments with cohesive ends, were purified by gel electrophoresis (2% agarose followed by NucleoSpin® gel and PCR Clean-up; Macherey Nagel) and used as an insert for ligation. PCR products were cloned in pSTARRseq-ori plasmid^4^(Addgene #71,509) which contains the *ccdB* negative selection gene, and was thus prepared in E. coli DB3.1 (ThermoFisher Scientific). The plasmid DNA was digested with the AgeI and SalI restriction enzymes (2 h 37 °C). Shrimp alkaline phosphatase (1 μL New England Biolabs, 1units/μL) was then added to the digested DNA to fully prevent self religation. The AgeI-SalI 2.5 kb fragment was gel-purified and used as a cloning vector. 60 ng of insert and 60 ng vector were ligated in 30 µL with T4-DNA ligase (New England Biolabs; 3 h room temperature) and the ligation product was directly introduced in NEB® 5-alpha Competent E. coli (High Efficiency; New England Biolabs) which were spread on 10 Petri dishes with LB agar + 100 μg/ml ampicillin. Antibiotic-resistant colonies (> 10 000) were grown overnight at 37 °C and then scraped in LB liquid medium. Bacteria were recovered by centrifugation for plasmid extraction (NucleoBond Xtra Midi kit; Macherey Nagel). DNA (100 to 150 μg) was dissolved in Tris 5 mM pH7.5 EDTA 0.1 mM.

### Plasmids and luciferase reporter constructs.

SV40 driven expression vectors were derived from the construct pSG5 (Agilent). pSG5-TRα1 and pSG5-TRβ1 respectively encode the murine and chicken T3 receptors. pSG5-RXRα encodes the human RXRα receptor. pGL2-DR4luc derives from pGL2-promoter (Promega). It contains a minimal SV40 promoter located downstream to a XhoI-BglII insert with 5 repeats of the 5′AGATCCAGGCAGGTCATTTCAGGACAGCCCAGATC3′ consensus DR4 sequence (underlined). Reporter constructs with luciferase reporter were derived from this construct, by replacing the XhoI-BglII by inserts prepared by PCR from the oligopool with specific primers containing these restriction sites.

### Plasmid libraries transfection

All transfection experiments were performed in the HEK293 cell line (ATCC : 293 [HEK-293] CRL-1573 ™) seeded in 6-well plates (10^5^ cells/well). Cells were transfected the day after seeding with plasmids and library DNA using the TransIT-Lt1 transfection reagent (Mirus Corporation, Madison WI, USA). Because the cells express endogenous receptors at very low level, expression vectors derived from pSG5 (Agilent Technology) were included in all experiments. We also included the pGL2-DR4luc reporter plasmid as an internal control. T3 (Sigma Aldrich) was added 6 h later (10^-8^ M final) and cells were harvested the following day for either luciferase activity measurement or RNA analysis. Luciferase activity was measured in cell lysates with the firefly luciferase assay system (Promega Madison WI, USA).

### RNA extraction and quality controls

RNAs were extracted from transfected cells using the NucleoSpin RNA kit (Macherey Nagel) according to manufacturer instruction, including the recommended step of RNase-free DNase I treatment on the extraction column. The complete elimination of contaminant DNA from purified RNA was achieved by repeating the same procedure. cDNAs were prepared from 1 μg of RNA (M-MLV reverse transcriptase, Promega) using random hexamers (Eurogentec) as primers in a final volume of 20 μL. Reactions were stopped by the addition of EDTA (1 mM final) and the products diluted (1/4) with water. A PCR reaction was performed using the library amplification primers to verify after agarose gel electrophoresis the amplification of 200-mers (library 1) or 150-mers (library 2) amplicons. The complete absence of contaminating DNA in the RNA preparations, which is indispensable for later analysis, was verified by performing a mock RT-qPCR in which the reverse transcriptase was omitted.

### Preparation of cDNA libraries for deep sequencing.

Illumina sequencing requires the initial identification of DNA clusters on flow cells, which fails if all sequences are identical at the 5′ extremity. In order to generate sequences diversity at the 5′ end of the amplicons, 3 independent PCR amplifications were performed for each cDNA starting from 2 μL of diluted sample.

Primers pair 1: 5′TCGTCGGCAGCGTCAGATGTGTATAAGAGACAGTCACTGGAGTTGTCCCAATTCTTG3′ 5′GTCTCGTGGGCTCGGAGATGTGTATAAGAGACAGCGTCGACGCTAGCTCTGCAT3′.

Primers pair 2: 5′TCGTCGGCAGCGTCAGATGTGTATAAGAGACAGNTCACTGGAGTTGTCCCAATTCTTG3′ 5′GTCTCGTGGGCTCGGAGATGTGTATAAGAGACAGNCGTCGACGCTAGCTCTGCAT3′.

Primers air 3: 5′TCGTCGGCAGCGTCAGATGTGTATAAGAGACAGNNTCACTGGAGTTGTCCCAATTCTTG3′ 5′GTCTCGTGGGCTCGGAGATGTGTATAAGAGACAGNNCGTCGACGCTAGCTCTGCAT3′.

The following annealing schedule was: 9 cycles for 95 °C 30 s, 68 °C for 30 s; 72 °C for 15 s It was followed by 24 cycles with a lower hybridization temperature: 95 °C for 30 s, 58 °C for 30 s; 72 °C for 15 s and a final elongation step at 72 °C for 7 min. The 3 PCR products corresponding to a single cDNA were pooled and purified after agarose gel electrophoresis, using NucleoSpin Gel and PCR Clean-up kits (Macherey–Nagel).

The sequence diversity was further increased by adding to each sample 10% of PhiX Control v3 library (Illumina) and DNA sequencing was performed on a Miseq sequencer (Illumina Microkit V2 300 cycles) using the Nextera V2 index kit (Illumina). Single end reads (> 0.9.10^6^ reads/library) were trimmed from adaptor and plasmid sequences before mapping to the mouse genome (GRCm38/mm10 version. Bowtie2; Galaxy Version 2.4.2). Reads which did not mapped only once (< 20%) were binned. Mapped data (.bam files) were converted to .bed files. Count tables were prepared by using BedTools^19^. The Intersect intervals function (Galaxy Version 2.31.1 + galaxy0) was used to identify overlaps between these BED files and the BED file corresponding to the transfected library, reporting the number of overlaps. DR4 elements were identified using FIMO^11^ (p-value < 10^–6^) (https://meme-suite.org/meme/doc/fimo.html) and the previously defined consensus matrix. Differential analysis was used to identify the fragments that mediate a T3 response (DESeq2, Galaxy Version 2.11.40.8 + galaxy0; 2 factors; T3 treatment and batch effect. Adjusted p-value < 0.05). The use of DESeq2 is only valid if a large fraction of the tested fragment is inactive. If a large fraction of active fragment are expected, it is advisable to use other methods and to introduce a number of internal control fragments in the library.

### Critical steps for SOSHI-seq

It is important to maintain the diversity of the library throughout the procedure. This requires the avoidance of extensive amplification of the oligopool (< 13 PCR cycles) and the extraction of DNA from a large number of bacteria colonies (> 10 000) without liquid culture. The transfection efficiency of HEK293 cells should also be high. The luciferase activity derived from the internal control plasmid pGL2-DR4luc enables to verify both the transfection efficiency and a robust T3 response of the cells (typically > 100 fold-change in luciferase activity). Western blotting should also demonstrate the abundance of TR and RXR. The complete absence of contamination DNA in the RNA preparations must be verified by a PCR test, as it would introduce a detrimental and variable background.

## Supplementary Information

Below is the link to the electronic supplementary material.

Supplementary Material 1.

Supplementary Material 2.

Supplementary Material 3.

Supplementary Material 4.

## Data Availability

Raw sequencing data are available at Gene Expression Omnibus (GSE298259).
