# Supplementary figures and images for: SOSHI-seq: a high-throughput screening assay to test the functionality of putative response elements for nuclear hormone receptors
Source: Sci Rep. 2025 Dec 6;16:436. doi: 10.1038/s41598-025-29970-8 (PMC12775020; doi:10.1038/s41598-025-29970-8)

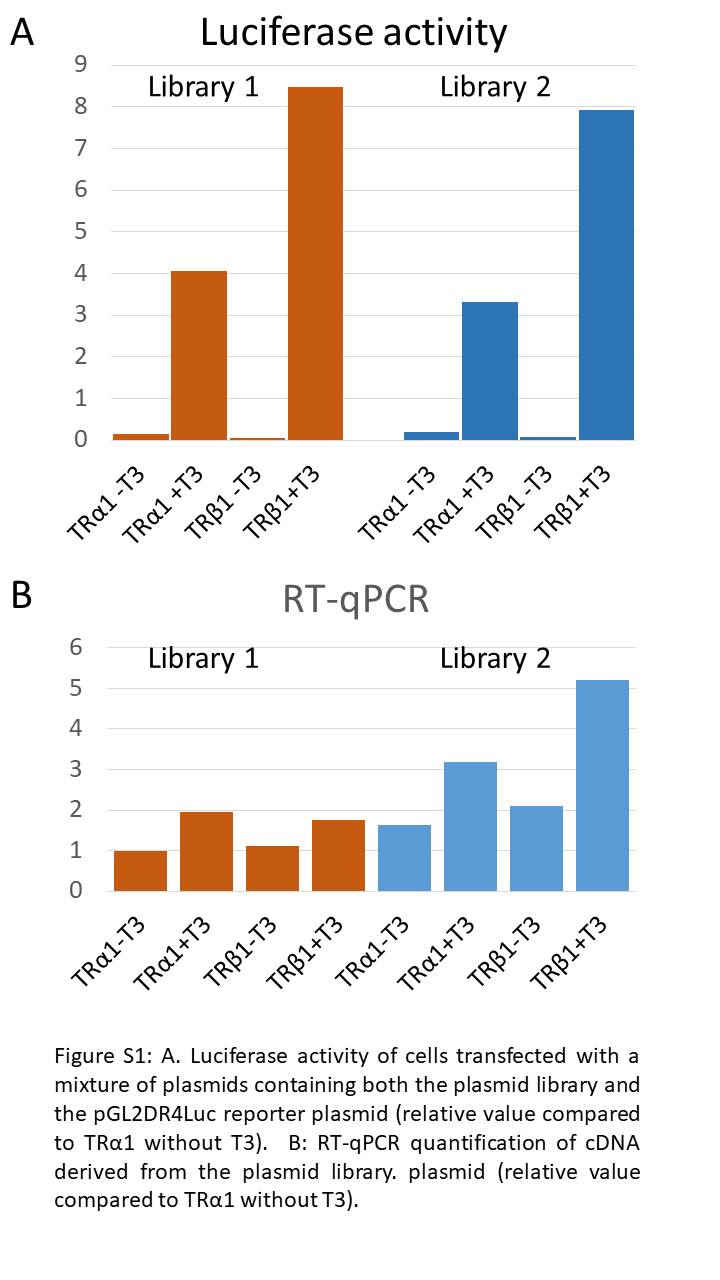

Supplement: Supplementary file 1 — Supplementary Material 1. [file 41598_2025_29970_MOESM1_ESM.tif]

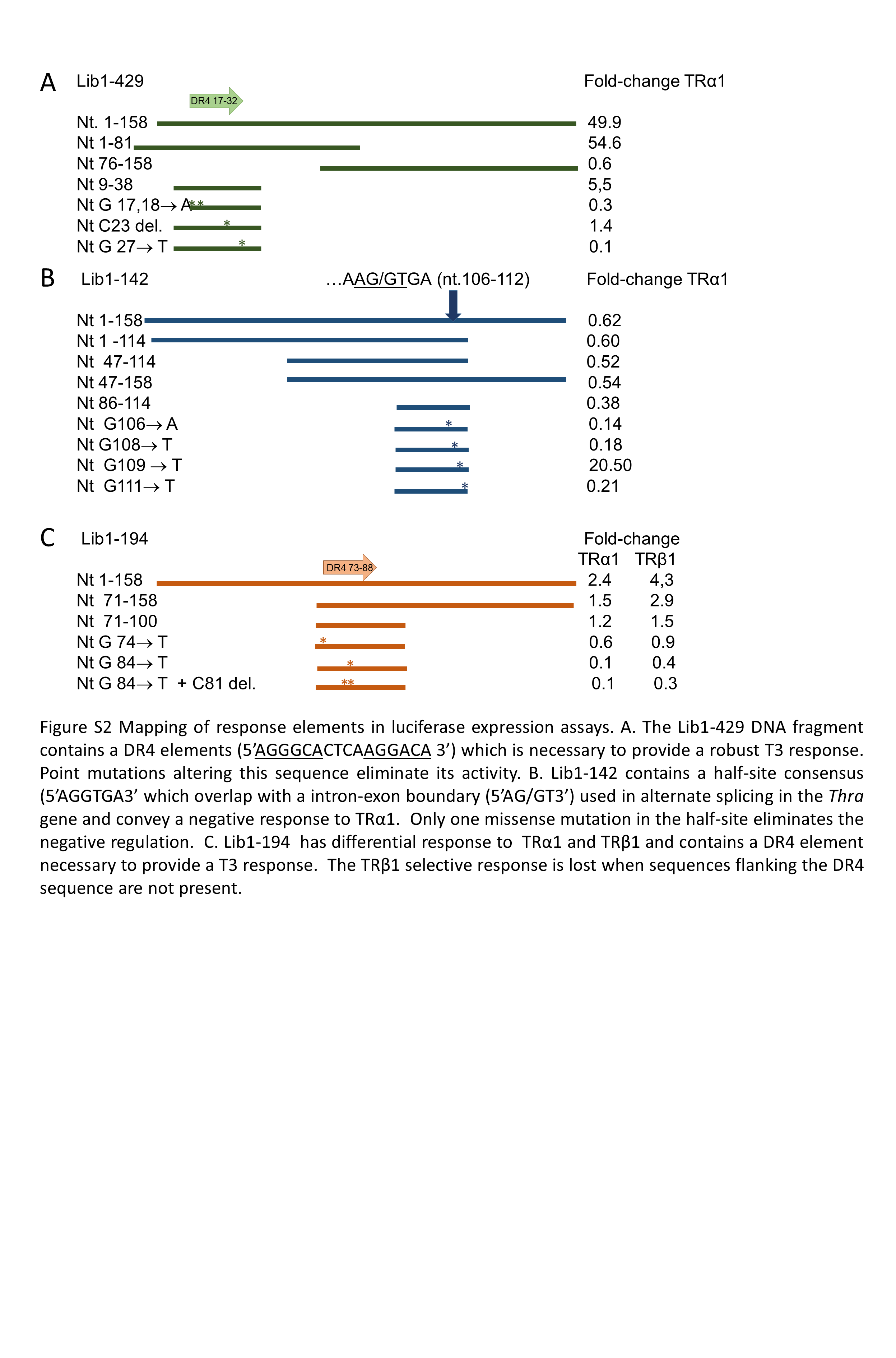

Supplement: Supplementary file 2 — Supplementary Material 2. [file 41598_2025_29970_MOESM2_ESM.tif]
